# Genetic Diversity of Merozoite Surface Protein-1 and -2 Genes in *Plasmodium falciparum* Isolates among Asymptomatic Population in Boset and Badewacho Districts, Southern Ethiopia

**DOI:** 10.1155/2022/7728975

**Published:** 2022-12-14

**Authors:** Tsegaye Chekol, Gezahegn Solomon Alemayehu, Weynshet Tafesse, Gudeta Legesse, Biruk Zerfu, Temesgen File, Mistire Wolde, Lemu Golassa

**Affiliations:** ^1^Department of Medical Laboratory Sciences, College of Health Sciences, Addis Ababa University, Addis Ababa, Ethiopia; ^2^Research and Community Service Center, College of Health Science Defense University, Bishoftu, Ethiopia; ^3^Department of Medical Laboratory Science, College of Medicine and Health Sciences, Wachamo University, Hossna, Ethiopia; ^4^Department of Medical Laboratory Science, College of Medicine and Health Sciences, Arsi University, Assela, Ethiopia; ^5^Department of Applied Biology, Adama Science and Technology University, Adama, Ethiopia; ^6^Aklilu Lemma Institute of Pathobiology, Addis Ababa University, Addis Ababa, Ethiopia

## Abstract

**Background:**

The genetic variation of *Plasmodium falciparum* has been studied to assess local malaria transmission genetic profile using evidence-based intervention measures. However, there are no known previous reports of *P. falciparum* polymorphism in Badewacho and Boset districts, Southern Ethiopia. The purpose of this study was to determine the genetic diversity of the merozoite surface protein-1 and -2 (*msp*-1 and *msp*-2) allelic families in *P. falciparum* isolates from an asymptomatic populations.

**Methods:**

This study was conducted from finger-prick blood samples spotted on 3 mm Whatman filter paper collected during a community-based cross-sectional study. Nested polymerase chain reaction amplification was used to type the allelic variants of *msp*-1 and *msp*-2.

**Results:**

From 669 asymptomatic study participants, a total of 50 samples positive for *P. falciparum* were included for molecular analysis. Of 50 positive samples, 43 *P. falciparum* isolates were successfully amplified for the *msp*-1 and *msp*-2 allelic families. A total of twelve different allele sizes (75–250 bp) were identified within the three allelic families of *msp*-1, whereas ten different allele sizes (250–500 bp) were detected within the two allelic families of *msp*-2. MAD20 had a higher allelic proportion, 65% among allelic families of *msp*-1, whereas the 3D7 allelic family 90.7% was higher in *msp*-2. A slightly higher frequency of polyclonal infection 53.5% was found in *msp*-2 allelic family, whereas a low proportion polyclonal infection 46.5% was found in *msp*-1 allelic family. The overall mean multiplicity of infection (MOI) for *msp*-1 and *msp*-2 was identical (MOI = 1.56). Correspondingly, the expected heterozygosity (He) value for *msp*-1 (He = 0.23) and *msp*-2 (He = 0.22) was almost similar.

**Conclusions:**

The findings of this study revealed low genetic diversity of the *msp*-1 and *msp*-2 allelic families in *P. falciparum* isolates. However, continued monitoring status of the local genetic diversity profile in the *P. falciparum* population is required to support current malaria control and elimination strategies.

## 1. Introduction

Malaria remains a major public health problem in the world. According to the World Malaria Report, an increase in malaria cases was observed between two consecutive years, and it is estimated that 241 million cases of malaria occurred worldwide in 2020, whereas 227 million cases in 2019. In 2020, with high burden of malaria, 95% of malaria cases and deaths originated from the World Health Organization (WHO) African Region [1]. The overall prevalence of malaria in Ethiopia was 0.5–1.2% by microscopy and/or malaria rapid diagnostic test based on the Ethiopia Malaria Indicator Survey [2].

Among human malaria parasites, *Plasmodium falciparum* is characterized by high genetic variation in several populations of malaria parasite isolates [3]. As a result, *P. falciparum* causes life-threatening disease and challenges the development of effective drugs, diagnostic tools, and vaccines in the global community [4]. Thus, understanding the genetic diversity of *P. falciparum* could support the current malaria control and elimination effort in the world [5]. There are several polymorphic gene markers in *P. falciparum* isolates, such as the merozoite surface protein-1 and -2 (*msp*-1 and *msp*-2) genes, the glutamate-rich protein (*glurp*) gene, and the circumsporozoite protein (*csp*) gene [6]. Among the genetic marker genes in *P. falciparum* isolates, the *msp*-1 and *msp*-2 genes are widely used to assess allelic diversity and play a vital role in determining the extent of malaria transmission in a given community [7].

Merozoite surface protein-1 (MSP-1) is a surface protein in *P. falciparum* isolates with a size of 190 kDa, encoded by the *msp*-1 gene found on chromosome 9. Among the seven variable blocks in *msp*-1 gene, block 2 is the most polymorphic region that occurs in three allelic families, such as K1, MDA20, and R033 [8, 9]. Merozoite surface protein-2 (MSP-2) is a glycoprotein in *P. falciparum* isolates, encoded by the *msp*-2 gene found on chromosome 2. Of the five variable blocks, block 3 is the most polymorphic central region with two allelic families, namely, FC27 and 3D7 [10, 11].

In the era of malaria control and elimination in the world, the continuous assessment of the genetic variation of *P. falciparum* by genotyping *msp*-1 and *msp*-2 is essential to monitor the extent of intervention in different scenarios of malaria [11–13]. In line with this, several studies have been carried out on the genetic variation of *P. falciparum* in symptomatic malaria patients in the world [14–18]. However, limited studies were found in Ethiopia [15, 19–21]. Furthermore, there are no known studies on the genetic polymorphism of *P. falciparum* from asymptomatic individuals in Boset and East Badewacho districts in Southern Ethiopia. This study aimed to assess the genetic variation of *msp*-1 and *msp*-2 in *P. falciparum* from asymptomatic individuals from Boset and East Badewacho districts, Southern Ethiopia.

## 2. Materials and Methods

### 2.1. Study Design and Area

This study was carried out from samples collected during a community-based cross-sectional study in Boset and East Badewacho districts from March to June 2020, Southern Ethiopia. Boset district is located in the East Shewa Zone in the Oromia Region of Southeastern Ethiopia. The altitude of the district varies from 1100 to 2700 m above sea level and receives an annual average of rainfall that varies between 700 and 800 mmHg. The district is characterized by a hot and dry climate with an average annual temperature that varies between 25 and 30°C for the tropical and between 15 and 20°C for the subtropical. It is an agricultural area, and Nura Hera of upper Awash agro-industry is found here, where extensive agriculture is carried out in Ethiopia through the irrigation of the Awash River. Badewacho district is located in Hadiya Zone in southern nation nationality population region, South Ethiopia. It is found 225 km from the capital city, Addis Ababa. The altitude of the district ranges from 1501 to 2500 m above sea level and receives a mean annual rainfall of 801–1400 mmHg. The average annual temperature ranges from 17.6 to 22.5°C. Agriculture is the principal source of livelihood for the rural population. Both districts are known to be malarial with decreased malaria transmission due to the current intervention measure.

### 2.2. Study Population and Blood Sample Collection

This study was carried out from samples collected during community-based studies in East Badewacho district (415 study participants) and Boset district (254 study participants). A total of 50 microscopy and polymerase chain reaction (PCR) confirmed *P. falciparum* samples, 37 dried blood spot samples from Badewacho district, and 13 from Boset district were included for molecular analysis of *msp*-1 and *msp*-2 allelic families in this study. Three to four drops of finger-pricked blood were spotted on 3 mm Whatman filter paper, dried, and placed in airtight plastic bags with desiccant and stored at −20°C in the Parasitology Research Laboratory at Aklilu Lemma Institute of Pathobiology, Addis Ababa University, Addis Ababa, Ethiopia.

### 2.3. Microscopy

The level of parasitemia was taken from microscopy blood film examination. The thin smear was used for malaria parasite species identification, whereas the thick smear was used to estimate parasite density according to the WHO protocol [22]. Parasite density was graded as very low (<100 parasites/*μ*l), low (100–499 parasites/*μ*l), moderate (500–4999 parasites/*μ*l), high (5000–10,000 parasites/*μ*l), and very high (>10,000 parasites/*μ*l).

### 2.4. Genomic DNA Extraction

Genomic DNA was extracted from dried blood spots using the Chelex–saponin method, and the final extracted genomic DNA samples were stored at −20°C until used for PCR amplification as described previously [23].

### 2.5. Allelic Typing of *msp*-1 and *msp*-2

Allelic typings were performed using a primer specific for the polymorphic regions of *P. falcipaurm msp*-1 (block 2) and *msp*-2 (block 3). Two round PCR amplifications were performed as described previously by Hamid et al. [14]. In the primary PCR reaction, primers span the whole genetic locus of *msp*-1 (block 2) and *msp*-2 (block 3), whereas the secondary/nested PCR reactions target the family specific alleles of *msp*-1 (K1, MAD20, and RO33) and *msp*-2 (FC27 and 3D7; Table 1). Both the primary and nested PCR reactions were performed in a final volume of 20 *μ*l containing 0.25 *μ*M of each primer and 1 unit of 5× hot fire pol master mix. In the primary PCR reaction, 4 *μ*l of DNA template was used, whereas in the secondary PCR reaction, 2 *μ*l of primary PCR products were used. The cycling conditions for the primary PCR were initial denaturation at 95°C for 3 minutes, followed by 35 cycles of denaturation at 95°C for 60 seconds; annealed at 58°C for 1 minute; extension at 72°C for 90 seconds; and a final extension at 72°C for 60 seconds and holding at 10° C. The cycling conditions for secondary PCR are the same as primary PCR except the number of cycles, 30 cycles for nested PCR. The PCR products were stored at 4°C until analysis. Five microliters of the amplified products were electrophoresed using 2% agarose gels made of Tris–borate–EDTA for *P. falciparum*. The PCR products were then stained with ethidium bromide for visual detection and estimation of the amplicon products with respective to 50 base pair DNA ladder by ultraviolet transilluminator light. The PCR works were carried out at Aklilu Lemma Institute of Pathobiology, Addis Ababa University.

### 2.6. Data Analysis

Data were entered and analyzed using SPSS version 25. The proportion of *msp*-1 and *msp*-2 allelic was calculated to present the distribution of different allelic families. Associations between proportions were tested using the chi-square test. The estimation of the multiplicity of infection (MOI) was performed using the average number of PCR fragments per infected individual. Genetic diversity of *Plasmodium falciparum* isolates was measured using expected heterozygosity (He). He was calculated using formula, He = *n*/(*n* − 1)(1−∑Pi2), where *n* is the number of isolates analyzed, and Pi is the frequency of each different allele at a locus. *P-*values ≤0.05 were considered to indicate statistical significance.

## 3. Results

### 3.1. Socio-Demographic Characteristics

A total of 43 microscopy and PCR-confirmed *P. falciparum*-positive isolates collected during the community-based study were included in this study. Of the 43 study participants, 55.8% (24/43) more men than women were enrolled. The mean age of the participants was 26.88 years (±15.78 SD), with a range of 2–80 years. The characteristics of the study participants are indicated in Table 2.

### 3.2. Allelic Diversity of *P. falciparum msp*-1 and *msp*-2

**Figure 1:**
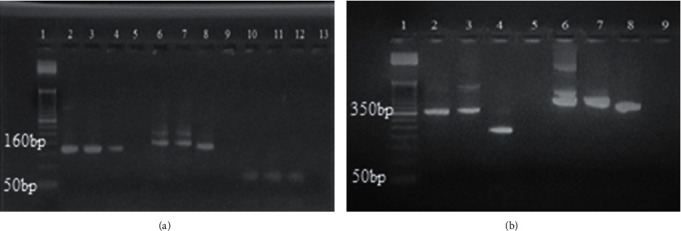
Gel electrophoresis of MSP allelic families. (a) Alleles of the *msp*-1 gene showing lane 1 (L1) 50 bp DNA ladder; positive samples: L2 and L3 for MAD20 (160 bp), L6 and L7 for K1 (200 bp), and L10 and L11 for RO33 (75 bp); positive control: L4, L8, and L12; negative control: L5, L9, and L13. (b) Alleles of the *msp*-2 gene showing lane 1 (L1) 50 bp DNA ladder; positive samples: L2 and L3 for FC27 (350 bp) and L6 and L7 for 3D7 (400 bp); positive control: L4 and L8; negative control: L5 and L9.

Of the 50 microscopy and PCR-confirmed *P. falciparum*-positive samples collected from the asymptomatic population, 43 *P. falciparum* isolates were successfully amplified for genetic diversity of *P. falciparum msp*-1 and *msp*-2 genes. Out of 43 *P. falciparum* isolates (Table 3), a total of 12 different allele sizes (75–250 bp) were identified within the three allelic families of *msp*-1 (MAD20, K1, and RO33) based on the size of the allelic fragments (Figure 1). MAD20 had a higher allelic proportion 65% (28/43), followed by K1, 46.5% (20/43), and RO33, 37.2% (16/43). A higher frequency of monoclonal infection 53.5% (23/43) was detected in *msp*-1 allelic families compared to polyclonal infection 46.5% (20/43) in a set of two or three specific allelic combinations. The overall mean MOI and He index for the *msp*-1 genotype were 1.56 and 0.23, respectively. Similarly, from 43 *P. falciparum* isolates, 10 different allele sizes (250–500 bp) were detected within the two allelic families of *msp*-2 (FC27 and 3D7). The frequency of 3D7 allelic family 90.7% (39/43) was highest compared with FC27 allelic family 62.8% (27/43) in *msp*-2. A lower frequency of monoclonal infection 46.5% (20/43) was detected in *msp*-2 allelic families compared with polyclonal infection 53.5% (23/43) in a set two alleles (FC27 and 3D7). The overall mean MOI and the He index for the *msp*-1 genotype were 1.56 and 0.22, respectively.

### 3.3. Allelic Family Profile of *msp*-1 and *msp*-2 across Age Groups, Sex, and Study Sites

All reported allelic families of *msp*-1 (MAD20, K1, and R033) and *msp*-2 (FC27 and 3D7) were identified among the isolates at the two study sites (Table 4). The distribution proportion of allelic families, namely, MAD20, K1, FC27, and 3D7, was higher in Badewacho district, whereas R033 was higher in Boset district. Statistically significant difference (chi-square test, *χ*^2^ = 19.89, *P* = 0.003) was observed between study sites only for the *msp*-1 allelic family. The distribution of both *msp*-1 and *msp*-2 specific allelic families with respect to age group and sex of the study participants showed a slight difference, but no statistically significant variation (*P* > 0.05) was observed.

**Figure 2:**
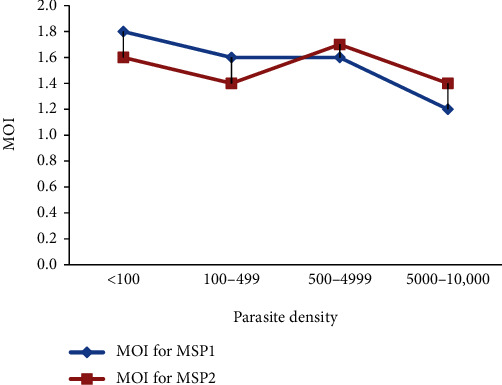
Relationship between multiplication of infection (MOI) and parasite density, parasite per *μ*l.

The MOI for *msp*-1 was higher in the younger and older age group, whereas the MOI for *msp*-2 showed a slight increase with increasing age, but no statistical difference was observed in both *msp*-1 (*P* = 0.268) and *msp*-2 (*P* = 0.607) allelic families. The MOI for the allelic families of *msp*-1 and *msp*-2 was higher in females, but the results did not show a significant variation in both *msp*-1 (*P* = 0.970) and *msp*-2 (*P* = 0.325). The MOI was higher in Bedawach district than Boset district; however, no statistical differences were observed in the allelic families of *msp*-1 (*P* = 0.436) and *msp*-2 (*P* = 0.385). Regarding parasite density, a decrease in MOI was shown as parasite density increased (Figure 2), except for parasite density, 500–4999 parasites per *μ*l; however, no statistical differences were observed in both *msp*-1 (*P* = 0.326) and *msp*-2 (*P* = 0.576).

## 4. Discussion

This study was carried out to assess the genetic diversity of the *msp*-1 and *msp*-2 genes in *P. falciparum* isolates collected from the asymptomatic population. In the present study, a low degree of genetic diversity, *msp*-1 (12 genotype) and *msp*-2 (10 genotype) allelic variants were found in *P. falciparum* population in Boset and East Badewacho districts, Southern Ethiopia.

In this study, of the three allelic families of *msp*-1 (MAD20, K1, and RO33), MAD20 was found to be the predominant polymorphic allele type. This pattern is consistent with studies from Adama in Ethiopia [24], East Africa [25], and Cameroon [26]. On the other hand, K1 and R033 were predominate in isolates in other studies from southwest Ethiopia [15], Nigeria [27], and Malaysia [17]. Regarding the two *msp*-2 allelic families, 3D7 were the most predominant compared with FC27 allelic families in this study. This is in line with reports from northeast Ethiopia [28], southwest Ethiopia [15], Burkina Faso [18], and Cameroon [29]. In contrast, the predominance of FC27 over the 3D7 allelic family was indicated in previous studies from northwest Ethiopia [21] and central Sudan [14]. This difference observed in the present study could be attributed to natural selection on the allelic family of *msp*-1 and *msp*-2 [30, 31]. Moreover, variation in the transmission setting, level of exposure, characteristics of study participants, and the method used for molecular genotype could be the cause of the discrepancy in the present study compared with the previous report.

In the present study, polyclonal infections for *msp*-1 allelic families, the overall mean MOI for both *msp*-1 and *msp*-2 (MOI = 1.56), and the expected He value for *msp*-1 (He = 0.23) and *msp*-2 (He = 0.23) were slightly lower in Boset and Badewacho districts. This finding is consistent with a report from Adama and its surroundings in Ethiopia [24], Djibouti [32], and Yemen [33]. In contrast, the MOI and He values reported in this study were lowest compared with others findings from southwest Ethiopia [15], northwest Ethiopia [21], Sudan [14], southwestern Nigeria [27], and Côte d'Ivoire [34]. The low MOI and He values in the study under report may be associated with the study area as the study cities are located under low transmission setting and the difference in the study participants [35, 36].

In the present study, no statistical association was observed between age group and MOI for both *msp*-1 and *msp*-2 allelic families, even if a slight difference were observed in the younger and older age group. This finding is similar to previous reports from northeast Ethiopia [28], Sudan [37], and Côte d'Ivoire [34]. In contrast, a statistically significant difference between MOI and age groups of the study participants was detected in other studies from Ghana [38], Nigeria [39], and Sudan [40]. Likewise, the MOI for *msp*-1 and *msp*-2 allelic families was higher in females, but without statistical significant variation. In this study, a decline in MOI was detected when parasite density increased, but no statistical differences were observed in both *msp*-1 and *msp*-2. This is compatible with studies from northwest Ethiopia [21] and Nigeria [41]. In contrast, association between MOI and parasite density has been reported in previous studies from Sudan [14], Senegal [42], and Congo [16]. This difference could be the development of acquired immunity with respect to the level of malaria transmission in the given area and the frequency of exposure to the malaria parasite [21, 43, 44].

Regarding the study sites, although no statistical differences were found between Badewacho and Boset districts, higher distribution of allelic families and MOI for both msp-1 and *msp*-2 was observed in Badewacho district. This could be explained partly by the difference in the sample size and degree of local malaria transmission. The use of small sample size and less sensitive molecular methods to differentiate minor fragments is the limitation of this study. However, as far as we know, the present study is the first action in Boset and Badewacho districts, which generates valuable information about the genetic diversity of polymorphic region of *msp*-1 and *msp*-2 allelic families in *P. falciparum* isolate.

## 5. Conclusion

In this study, lower genetic diversity of *msp*-1 and *msp*-2 allelic families in terms of MOI, He, and multiclonal infections was found in *P. falciparum* population in Boset and Badewacho districts, Southern Ethiopia. This study can be used as baseline data to assess the intensity of malaria transmission and evaluate the current malaria control and elimination programs in Ethiopia. Further study with a large sample size between these two study regions/sites will be required to analyze the genetic similarity and difference of circulating polymorphic marker of the *msp*-1 and *msp*-2 allelic families in *P. falciparum* isolates. In addition, investigation to differentiate minor fragments of *msp*-1 and *msp*-2 should consider the use of sensitive molecular laboratory methods, such as capillary electrophoresis, single nucleotide polymorphism, and next-generation sequencing.

## Figures and Tables

**Table 1:** Sequences of the primers for *msp*-1 and *msp*-2 genes in *Plasmodium falciparum* isolates.

Locus	PCR round	Primer	Primer sequences^a^
MSP-1 (block 2)	Primary PCR	msp1-F1	5′-CTA GAA GCT TTA GAA GAT GCA GTA TTG-3′
		msp1-R1	5′-CTT AAA TAG TAT TCT AAT TCA AGT GGA-3′
		K1-F	5′-AAT GAA GAA GAA ATT ACT CA AAA GGT-3′
		K1-R	5′-GCT TGC ATC AGC TGG AGG GCT TGC ACC-3′
	Secondary PCR	MAD20-F	5′-AAA TGA AGG AAC AAG TGG AAC AGC TGT-3′
		MAD20-R	5′-ATC TGA AGG ATT TGT ACG TCT TGA ATT-3′
		R033-F	5′-TAA AGG ATG GAG CAA ATA CTC AAG TTG-3′
		R033-R	5′-CAT CTG AAG GAT TTG CAG CAC CTG GAG-3′
Msp-2 (block 3)	Primary PCR	msp2-F1	5′-ATG AAG GCA ACT AAA ACA TTG TCT ATT-3′
		msp2-R1	5′-CTT TGT TAC CAT CGG TAC ATT CTT-3′
		3D7-F	5′-GCA GAA AGT AAG CCT TCT ACT GGT GCT-3′
	Secondary PCR	3D7-R	5′-GAT TTG TTT CGG CAT TAT TAT-GA-3′
		FC27-F	5′-GCA AAT GAA GGT TCT AAT ACT AAT AG-3′
		FC27-R	5′-GCT TTG GGT CCT TCT TCA GTT GAT TC-3′

^a^As described previously by Khaireh et al. [12] and Hamid et al. [14].

**Table 2:** Socio-demographic characteristics of the study participants, Boset and Badewacho districts, Southern Ethiopia.

Study participants (*N* = 43)		
Variables		Number (%)
Sex	Male	24 (55.8)
	Female	19 (44.2)
Age group (years)	<5	3 (7.0)
	5–14	7 (16.3)
	15–24	8 (18.6)
	25–34	14 (32.6)
	>34	11 (25.6)
Marital status	Single	16 (37.2)
	Married	26 (60.5)
	Divorced	1 (2.3)

**Table 3:** Genetic diversity of *msp*-1 and *msp*-2 in *Plasmodium falciparum* population in Boset and Badewacho districts, Southwest Ethiopia.

Gene (*N* = 43)	Type of alleles	Frequency of positive isolates, *n* (%)	Alleles size (bp)	No. of observed alleles	Overall MOI	HE
*msp*-1	MAD20	10 (23.3)	160–250	5	1.56	0.23
	K1	5 (11.6)	100–200	3		
	RO33	8 (18.6)	75–200	4		
	MAD20 + K1	12 (27.9)				
	MAD20 + RO33	5 (11.6)				
	K1 + RO33	2 (4.7)				
	MAD20 + K1 + RO33	1 (2.3)				
*msp*-2	FC27	4 (9.3)	250–450	5	1.56	0.22
	3D7	16 (37.2)	300–500	5		
	FC27 + 3D7	23 (53.5)				

**Table 4:** Distribution of *Plasmodium falciparum msp*-1 and *msp*-2 allelic family profile by age groups, sex, and study site.

Gene (*N* = 43)	Type of alleles	Age group (years), *n* (%)					Sex, *n* (%)		Study site, *n* (%)	
		<5	5–14	15–24	25–34	>34	Male	Female	Badewacho	Boset
*msp*-1	MAD20	1 (10)	1 (10)	2 (20)	4 (40)	2 (20)	6 (60)	4 (40)	9 (90)	1 (10)
	K1	0 (0)	0 (0)	1 (20)	3 (60)	1 (20)	3 (60)	2 (40)	4 (80)	1 (20)
	RO33	0 (0)	2 (25)	1 (12.5)	3 (37.3)	2 (25)	4 (50)	4 (50)	2 (25)	6 (75)
	MAD20 + K1	2 (16.7)	2 (16.7)	3 (25)	1 (8.3)	4 (33.3)	6 (50)	6 (50)	12 (100)	0 (0)
	MAD20 + RO33	0 (0)	2 (40)	1 (20)	2 (40)	0 (0)	3 (60)	2 (40)	2 (40)	3 (60)
	K1 + RO33	0 (0)	0 (0)	0 (0)	1 (50)	1 (50)	2 (100)	0 (0)	2 (100)	0 (0)
	MAD20 + K1 + RO33	0 (0)	0 (0)	0 (0)	0 (0)	1 (100)	0 (0)	1 (100)	1 (100)	0 (0)
	Chi-square, *χ*^2^			16.385			3.263			19.89
	*P*-value			0.875			0.775			0.003
	MOI for *msp*-1	1.7	1.6	1.5	1.4	1.8	1.5	1.6	1.5	1.4
	*P*-value			0.268			0.970			0.436
*msp*-2	FC27	1 (25)	0 (0)	0 (0)	2 (50)	1 (25)	2 (50)	2 (50)	4 (100)	0 (0)
	3D7	0 (0)	5 (31.3)	4 (25)	4 (25)	3 (18.8)	10 (62.5)	6 (37.5)	9 (56.3)	7 (43.8)
	FC27+ 3D7	2 (8.7)	2 (8.7)	4 (17.4)	8 (34.8)	7 (30.4)	12 (52.2)	11 (47.8)	19 (82.6)	4 (17.4)
	Chi-square			9.05			0.468			4.96
	*P*-value			0.338			0.791			0.084
	MOI for *msp*-2	1.3	1.3	1.5	1.6	1.8	1.5	1.7	1.6	1.4
	*P*-value			0.607			0.325			0.385

## Data Availability

The data generated and analyzed during this study are included in this research manuscript, and additional data can be obtained from the corresponding author upon request.
